# Decreased Th1-Type Inflammatory Cytokine Expression in the Skin Is Associated with Persisting Symptoms after Treatment of Erythema Migrans

**DOI:** 10.1371/journal.pone.0018220

**Published:** 2011-03-31

**Authors:** Johanna Sjöwall, Linda Fryland, Marika Nordberg, Florence Sjögren, Ulf Garpmo, Christian Jansson, Sten-Anders Carlsson, Sven Bergström, Jan Ernerudh, Dag Nyman, Pia Forsberg, Christina Ekerfelt

**Affiliations:** 1 Division of Infectious Diseases, Department of Clinical and Experimental Medicine, Faculty of Health Sciences, University of Linköping, Linköping, Sweden; 2 Division of Clinical Immunology, Department of Clinical and Experimental Medicine, Faculty of Health Sciences, University of Linköping, Linköping, Sweden; 3 Division of Dermatology, Department of Clinical and Experimental Medicine, Faculty of Health Sciences, University of Linköping, Linköping, Sweden; 4 Department of Clinical Microbiology, Kalmar Hospital, Kalmar, Sweden; 5 Department of Microbiology, University of Umeå, Umeå, Sweden; 6 The Åland Borrelia group, the Åland Islands, Finland; University of Sao Paulo, Brazil

## Abstract

**Background:**

Despite the good prognosis of erythema migrans (EM), some patients have persisting symptoms of various character and duration post-treatment. Several factors may affect the clinical outcome of EM, *e.g.* the early interaction between *Borrelia* (*B.*) *burgdorferi* and the host immune response, the *B. burgdorferi* genotype, antibiotic treatment as well as other clinical circumstances. Our study was designed to determine whether early cytokine expression in the skin and in peripheral blood in patients with EM is associated with the clinical outcome.

**Methods:**

A prospective follow-up study of 109 patients with EM was conducted at the Åland Islands, Finland. Symptoms were evaluated at 3, 6, 12 and 24 months post-treatment. Skin biopsies from the EM and healthy skin were immunohistochemically analysed for expression of interleukin (IL)-4, IL-10, IL-12p70 and interferon (IFN)-γ, as well as for *B. burgdorferi* DNA. Blood samples were analysed for *B. burgdorferi* antibodies, allergic predisposition and levels of systemic cytokines.

**Findings:**

None of the patients developed late manifestations of Lyme borreliosis. However, at the 6-month follow-up, 7 of 88 patients reported persisting symptoms of diverse character. Compared to asymptomatic patients, these 7 patients showed decreased expression of the Th1-associated cytokine IFN-γ in the EM biopsies (p = 0.003). *B. afzelii* DNA was found in 48%, *B. garinii* in 15% and *B. burgdorferi* sensu stricto in 1% of the EM biopsies, and species distribution was the same in patients with and without post-treatment symptoms. The two groups did not differ regarding baseline patient characteristics, *B. burgdorferi* antibodies, allergic predisposition or systemic cytokine levels.

**Conclusion:**

Patients with persisting symptoms following an EM show a decreased Th1-type inflammatory response in infected skin early during the infection, which might reflect a dysregulation of the early immune response. This finding supports the importance of an early, local Th1-type response for optimal resolution of LB.

## Introduction

Following a bite by a tick infected with *Borrelia* (*B.*) *burgdorferi*, the innate immune system is the first line of defense to encounter the spirochetes. This initial event is likely to determine the magnitude and quality of both the innate and the ensuing adaptive response [1]. Erythema migrans (EM) is by far the most common symptom of Lyme borreliosis (LB) worldwide [2]–[4]. In general, the prognosis of antibiotic-treated EM is good [5]–[7]. However, some patients are troubled by persisting symptoms of various duration and character post-treatment [7], [8]. Follow-up studies on adult patients with disseminated infection, *i.e.* neuroborreliosis (NB), indicate that as many as 50% of the patients suffer from persisting complaints after antibiotic treatment [9]–[12].

The reasons for differences in the clinical outcome of LB are probably multi-factorial, albeit, largely unknown. To date, there is no convincing evidence that symptoms post-treatment would be caused by a persisting infection [13]–[15]. On the contrary, the symptoms may be due to an improperly regulated immune response to *B. burgdorferi*, *i.e.* a weak initial inflammatory response, not generating proper down-regulatory feedback mechanisms. Indeed, previous investigations in both mice [16], [17] and humans [18]–[21] have indicated that a successful clinical outcome requires an effective inflammatory T helper cell (Th) 1-type response that is adequately balanced by an anti-inflammatory and Th2-like response against *B. burgdorferi*.

Previous studies have shown a predominant Th1-type inflammatory response in the skin of EM lesions [1], [22]. Furthermore, analysis of cytokine mRNA in EM skin biopsies from patients without concomitant EM-associated symptoms, have shown expression of both IFN-γ and IL-10, whereas cytokine analysis in skin of EM-patients with such symptoms have additionally shown a large number of macrophage-derived pro-inflammatory cytokines, such as TNF-α, IL-1β and IL-6 [23]. This indicates that cytokine expression patterns in the skin may be of importance for the development of symptoms. However, very little is known about the clinical outcome of EM in relation to early cytokine expression *in situ* in infected skin, which constitutes the primary entrance route of the spirochetes.

Our aim was to ascertain whether differences in early local cytokine responses are associated with the clinical outcome of EM. Therefore, we analysed cytokine expression *in vivo* in human EM-skin biopsies in relation to clinical follow-up data. We also investigated other factors that might influence the outcome, including the genotype of the infecting *B. burgdorferi* species and allergic predisposition in the host.

## Results

All patients, except one, completed the antibiotic treatment without disruption. This patient, who was asymptomatic at all follow-up time points, discontinued the treatment after seven days, due to a suspected allergic reaction.

Eighteen of the 109 patients originally included in the study were excluded due to missing data from the 6-month follow-up. Ten of the remaining 91 reported persisting symptoms at the 6-month follow-up; three of those were excluded due to presence of symptoms >3 weeks before inclusion, resulting in a total of 88 patients (55 women, 33 men, median age 57 years), seven (8%) of whom reported persisting symptoms 6 months post-treatment. The symptoms included: arthralgia in knee (2/7), elbow (2/7), wrist (1/7), ankle (1/7), fatigue (1/7), back pain (1/7) and hypoesthesia in the skin close to the previous site of the EM (1/7), whereof five still had symptoms at the 12 month follow-up and three 24 months post-treatment. However, none of them were re-treated with antibiotics due to the persisting symptoms. Besides one patient receiving doxycycline, all symptomatic patients were treated with a completed course of amoxicillin and none reported diseases or medication, which might have an impact on the immune system. No signs of disseminated infection, *i.e.* arthritis, NB or acrodermatitis chronicum atrophicans were observed during the study period. At the time of inclusion, the median duration of EM was 6 days (range 0–215 days). Three patients had multiple EM, whereof none had symptoms at the 6-month follow-up. Fifty-eight patients (66%) had observed a preceding tick-bite. No statistically significant differences in clinical characteristics, *B. burgdorferi* serology or allergic trait were found between the symptomatic and asymptomatic patients (Table 1).

**Table 1:** Clinical characteristics of the included patients.

Variable	Patients with symptoms at 6 mos *Included in IHC* (A)	Patients with no symptoms at 6 mos (B)	Statistical comparison (A vs B)	Patients with no symptoms at 6 mos *Included in IHC* (C)	Statistical comparison (A vs C)	In total (A+B)
**N**	7	81		18		88
**Sex F/M**	5/2	50/31	NS	11/7	NS	55/33
**Age (years) range**	30–68	23–88		23–73		23–88
**Age (years) median**	55	58	NS	49	NS	57
**Single/Multiple EM**	7/0	78/3	NS	16/2	NS	85/3
**Tick bite reported Y/N**	3/4	55/25	NS	14/4	NS	58/29
**EM size (cm) range**	5–30	3–37		5–16		3–37
**EM size (cm) median**	15	10	NS	9	NS	10
**Borrelia antibodies pos/n analysed**						
**C6**						
**3 mos**	4/7	39/81	NS	9/18	NS	43/88
**6 mos**	4/7	36/78	NS	7/18	NS	40/85
**IgM**						
**3mos**	7/7	48/81	NS	11/18	NS	55/88
**6mos**	6/7	43/78	NS	10/18	NS	49/85
**IgG**						
**3mos**	5/7	51/81	NS	14/18	NS	56/88
**6mos**	7/7	46/78	NS	12/18	NS	53/85
**Allergy Y/N**	1/6	25/56	NS	7/11	NS	26/62
**EM duration (days)** *						
**Range**	2–215	0–63		0–45		0–215
**Median**	4	6	NS	7	NS	6

*Duration of EM prior to antibiotic treatment.

Clinical characteristics of the included patients, statistical comparison of patients with and without symptoms at 6 months post-treatment, and data on patients who were asymptomatic at 6 months and were included in the immunohistochemical analysis of in vivo cytokine expression in EM skin biopsies. All patients are included in the column farthest to the right.

IHC, immunohistochemistry; F, female; M, male; EM, erythema migrans; Y, yes; N, no; pos, positive; Ig, immunoglobulin; NS, not significant.

**Figure 1:**
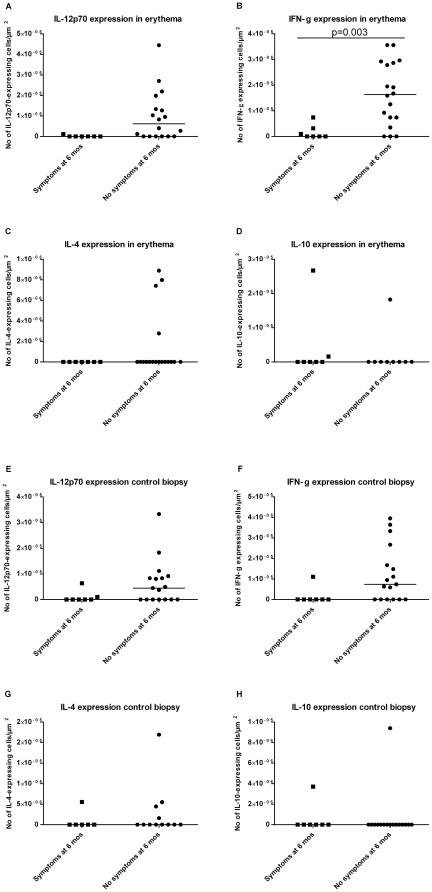
Immunohistochemical detection of cytokine expression in skin biopsies. Erythema migrans (EM) skin lesions and biopsies from unaffected skin of seven patients with and 18 patients without symptoms 6 months post-treatment. The diagrams illustrate the expression of interleukin (IL)-12p70 (A), interferon (IFN)-γ (B), IL-4 (C), and IL-10 (D) in EM-biopsies and IL-12p70 (E), IFN-γ (F), IL-4 (G), and IL-10 (H) in unaffected skin. Mann-Whitney U-test and a manual Bonferroni correction were used for statistical analyses and p≤0.0125 was considered significant.

The expression of IFN-γ was decreased in EM biopsies from symptomatic compared to asymptomatic patients (p = 0.003, Figure 1 B). In addition, the symptomatic patients showed a strong tendency for decreased expression of IL-12p70 in the EM lesions compared to the asymptomatic patients (p = 0.013, figure 1 A). The expression of IFN-γ was correlated with IL-12p70 in both EM and control biopsies (p≤0.01 for both, rho = 0.687 and rho = 0.771, respectively). The two patient groups did not differ regarding expression of IL-4 and IL-10 either in EM (Figure 1 C, D) or control biopsies (Figure 1 G, H). Neither did the expression of IL-12p70 and IFN-γ in unaffected skin differ between the two patient groups. The cytokine expressions did not differ between EM and unaffected skin (data not shown).

Determination of systemic cytokine levels were not carried out on the 12- and 24-month blood samples, because samples were missing in many patients. No statistically significant differences in cytokine levels in either plasma or serum or correlations of systemic cytokine levels with cytokine expression in skin biopsies were found between the two groups at any of the three points (at inclusion, at three or six months post-treatment) (Table 2).

**Table 2:** Systemic cytokine levels estimated by detection in multiple bead assays.

Time point	Cytokine	No. of samples with cytokine detectable^a^	Median (pg/ml)	Range (pg/ml)	Time point	Cytokine	No. of samples with cytokine detectable^a^	Median (pg/ml)	Range (pg/ml)	Statistical analysis
**Patients with no symptoms at 6 months (n = 80)**					**Patients with symptoms at 6 months (n = 7)**					
**Inclusion serum**	**IL–4**	**44/80**	0.32	0.065–42	**Inclusion serum**	**IL–4**	**6/7**	1.2	0.32–40.6	NS
	**IL–10**	**76/80**	7.72	0.32–180		**IL–10**	**7/7**	5.015	0.64–131	NS
	**IL–12p70**	**27/80**	0.32	0.065–1068.8		**IL–12p70**	**5/7**	1.43	0.32–21.59	NS
	**IFN–γ**	**33/80**	0.32	0.32–115.4		**IFN–γ**	**3/7**	0.32	0.32–12.74	NS
**Patients with no symptoms at 6 months (n = 60)**					**Patients with symptoms at 6 months (n = 5)** b					
**3 months plasma**	**IL–4**	**55/60**	6.1	0.065–69.35	**3 months plasma**	**IL–4**	**4/5**	9.63	0.32–22.5	NS
	**IL–10**	**59/60**	13.44	0.065–1221.4		**IL–10**	**5/5**	20.4	8.2–33	NS
	**IL–12p70**	**34/60**	0.36	0.065–1693		**IL–12p70**	**4/5**	1.98	0.065–16.3	NS
	**IFN–γ**	**56/60**	4.5	0.065–100.4		**IFN–γ**	**5/5**	2.36	0.64–10.56	NS
**6 months plasma**	**IL–4**	**55/60**	7.2	0.32–86.5	**6 months plasma**	**IL–4**	**2/5**	0.32	0.32–9.4	NS
	**IL–10**	**60/60**	13.13	2.36–1475.45		**IL–10**	**5/5**	10.69	2.14–16.88	NS
	**IL–12p70**	**25/60**	0.32	0.065–44		**IL–12p70**	**2/5**	0.065	0.065–8.52	NS
	**IFN–γ**	**57/60**	4.58	0.065–48.7		**IFN–γ**	**2/5**	0.32	0.065–4.87	NS

IL, interleukin; IFN, interferon; NS, not significant.

a Specified cytokine detectable within the range of the standard curve concentrations; concentrations below the lowest point of the standard curve were considered undetectable.

b Two samples missing, hence only 5/7 patients.

Seventy-five (81.5%) of the 92 EM biopsies, which were initially screened, were *B. burgdorferi* DNA positive (Table 3). Sequencing of the amplicons (n = 75) revealed 36 (48%) *B. afzelii*, 11 (15%) *B. garinii* and one (1%) *B. burgdorferi* sensu stricto (s.s.). In the initial screening of 87 control biopsies from unaffected skin, 20 (23%) were found to contain *B. burgdorferi* DNA. However, these findings could not be confirmed by subsequent sequencing. No differences in detection rates of *B. afzelii* or *B. garinii* DNA were observed between the symptomatic and asymptomatic patients (Table 3).

**Table 3:** PCR analysis of *B. burgdorferi* DNA in erythema migrans skin biopsies.

Variable	Patients with symptoms at 6 mos *Included in IHC* (A)	Patients with no symptoms at 6 mos (B)	Statistical comparison (A vs B)	Patients with no symptoms at 6 mos *Included in IHC* (C)	Statistical comparison (A vs C)	All included in the study (A+B)
**n (patients)**	7	81		18		88
**n (EM biopsies)**	7	85		20		92
**PCR positive**	6	42		11		48
**Subtypes** a ***B.afzelii*** **/** ***garinii*** **/** ***sensu stricto***	5/1/0	31/10/1	NS	6/5/0	NS	36/11/1
**Undetectable** a	1	43		9		44

a Includes both PCR positive biopsies in, which *B. burgdorferi* subtypes were undetectable (n = 27), and PCR negative biopsies (n = 17).

n, number; EM, erythema migrans; IHC, Immunohistochemistry; *B.*, *Borrelia*; NS, not significant.

## Discussion

We found that the local expression of the Th1-type cytokine IFN-γ was decreased in EM lesions from patients with persisting symptoms 6 months post-treatment in comparison with patients with no symptoms. In addition, a tendency for decreased expression of IL-12p70 was found in EM lesions from patients with persisting symptoms in comparison with asymptomatic patients. Furthermore, this finding is in line with the observed correlation between the expression of IFN-γ and IL-12p70, since IL-12p70 induces and maintains IFN-γ secretion, which validates the findings. Surprisingly, no differences in cytokine expression between the EM and unaffected control biopsies were found. These results support the hypothesis that a strong Th1-type response is required early in the course of infection to ensure a favorable outcome of LB. Our results are the first to show a possible association between clinical outcome and early, local cytokine expression in the skin at the site of EM.

The hypothesis that a strong, rapid Th1-type response is essential for a positive outcome of LB originates from the demand of a fine-tuned regulation of the immune system for establishment of an optimal immune response to a pathogen. A powerful, correct immune activation must be established early in the course of an infection and later, on a proper moment, needs to be down-regulated, in order to avoid chronic inflammation and subsequent tissue damage. A down-regulation, by secretion of immunosuppressive cytokines, will appear as a feed-back mechanism, provided that the initial immune response is strong enough [24].

Regarding LB, the importance of this regulation has previously been shown in experimental *B. burgdorferi* infection in mice by Bockenstedt's group [17]. Mice, that failed to eradicate the spirochetes, and developed clinical signs of infection (arthritis), showed weak Th1-type responses initially in the infection, with consequent lack of cytokine down-regulation and a gradual increase in the Th1-response instead. In contrast, resistant mice, who did eradicate the spirochetes without showing any clinical signs of infection, showed initially strong Th1-responses, that were subsequently down-regulated by an ensuing Th2-response. In man, the situation is more complex, since patients are treated with antibiotics, which most likely eradicate the spirochetes and modulate the immune response. Our hypothesis is that an early and strong Th1-response, besides providing an effective eradication, which is important in order to avoid dissemination before antibiotic treatment, is a prerequisite for a proper down regulation of the inflammation, thus avoiding low grade chronic inflammation that may cause persistent symptoms despite absence of the spirochetes. This hypothesis is corroborated by research findings regarding cytokine patterns in blood [20], [25] and in the target sites of disseminated LB, *i.e.* the cerebrospinal [26]–[28] and synovial [29], [30] fluids.

Overall, the long-term clinical outcome after treatment of EM was good, which agrees with earlier observations [31]–[34]. Indeed, among the seven patients with residual symptoms at the 6-month follow-up, all but three had recovered at the 2-year follow-up. The symptoms reported by the patients 6 months post-treatment are also known to occur in the general population [35] and have in addition been observed after other acute illnesses, such as Epstein-Barr virus infection [36].

One of the symptomatic patients had a very long EM duration (215 days). Skin lesions with long duration is, however, not uncommon in European EM, which are more commonly caused by *B. afzelii* and characterized by slowly expanding skin lesions and less associated symptoms at diagnosis compared to North American EM [23], [37]. This particular EM measured 30 cm in diameter and had a characteristic appearance. She presented with fatigue and arthralgia at baseline and gradually developed arthralgia in the elbow, without clinical signs of disseminated disease. The symptoms had disappeared spontaneously at the 24 month follow-up. *B. burgdorferi* serology was positive at baseline and at the follow-ups. Interestingly, the PCR assay was negative, which could be due to a spontaneous eradication of the spirochetes prior to treatment or a low density of spirochetes in the large erythematous area.

Allergic predisposition was assessed, because it has been shown that experimental Th2-deviation in mice leads to a less favorable outcome of LB [16]. However, we found no differences in allergic traits between symptomatic and asymptomatic patients. Furthermore, neither the development of symptoms, nor the disparities in Th1-type cytokine expression in EM-biopsies could be coupled to infection with different *B. burgdorferi* species, since the two patient groups did not differ with respect to the presence of *B. afzelii* or *B. garinii* infection. *B. afzelii* was the most common genotype found in this study, detected in 48% of the EM-biopsies, which concurs with previous European studies [38], [39]. In 18.5% of the EM biopsies, no *B. burgdorferi* DNA was detected.

The dissimilarities between the two groups regarding the expression of IFN-γ in the skin cannot be explained by differences in preparation of the specimens, because all staining and biopsy management were performed in the same manner, and the cytokine expressions were analysed randomly and blindly throughout the study.

The ability to establish Th1 or Th2 responses in mice is known to depend on the genetic background [17] and similar differences in humans have been reported [40], [41]. The observed differences between individuals in cytokine expression in the skin may thus be due to differences in the tendency to preferentially mount Th1 responses based on differences in immune genes. In line with this and according to our hypothesis that an early strong Th1-response is beneficial in *B. burgdorferi* infection, genetic differences may possibly also explain the different outcomes of EM. In any case, due to the non-experimental design of this study, the results must be interpreted with caution. However, since the two groups showed no differences in confounding factors, such as clinical or EM characteristics, the decreased expression of Th1-type cytokines in the EM biopsies from patients with persisting symptoms indicates that the local Th1-type response itself may contribute to the clinical outcome.

This study has, however, several limitations. We did not include a control group of patients without LB and the assessment of symptoms was merely based on questionnaires and phone interviews, without performing medical examinations or using validated tests for assessment of symptom severity. Neither was testing for co-infections with other tick-borne pathogens done. In addition, several patients were excluded due to lack of compliance with the follow-ups, which might partly be explained by a successful outcome in most patients and possibly by logistics in the isles.

In conclusion, patients with persisting symptoms 6 months after antibiotic treated EM showed reduced expression of Th1-like cytokines in the EM lesions prior to antibiotic treatment. This finding indicate an impact of the initial local immune response on the long-term outcome of LB and support the hypothesis that an early and strong Th1-type response is important for optimal resolution of LB. Additional studies are, however, needed to further substantiate our findings.

## Materials and Methods

### Ethics statement

The study was approved by The Regional Ethics Committee at Åland Central Hospital, the Åland Islands, Finland, on June 3, 2002. Written, informed consent was obtained from all participants.

### Study design

This prospective follow-up study was carried out from 2002 to 2004 on the Åland Islands, Finland, where LB is hyperendemic.

### Patients and blood samples

The study comprised 109 patients (72 women, 37 men; mean age 56 years) who had a newly discovered EM and were receiving care at Åland Central Hospital, Finland. Inclusion criteria were age ≥18 years and presence of an EM, defined as an expanding rash >5 cm in diameter. None of the included patients had received prior antibiotic treatment for the current EM. The participants completed a questionnaire with the following information: age, gender, general health status, other diseases that may have an influence on the immune system treatment with immunosuppressive drugs or antibiotics at inclusion or within two months prior to inclusion, symptoms at baseline, EM location and duration, number and size of the EM-lesions and whether a preceding tick-bite had been noticed. Patients gave blood samples at baseline and after 3, 6, 12 and 24 months and they answered the health questionnaire at the same time points, in some cases by telephone. Patients were divided into two groups at the 6-month follow-up: those with and those without self-reported symptoms, which had arisen in conjunction with the EM or afterwards, and could possibly be associated with the EM. The limit for debut of symptoms was set at <3 weeks prior to inclusion. The 6-month evaluation limit was chosen, because by that time symptoms consistent with both non-infectious complications and treatment failure with dissemination of the infection should have appeared. The patients were treated with amoxicillin (1 g twice daily), or in case of penicillin allergy with doxycycline (200 mg once daily), for 14 days.

### Procedures

*B. burgdorferi*-specific antibodies were measured in serum at all follow-up time points. ELISA kits were used to measure IgM and IgG antibodies against *B. burgdorferi* flagellum (Dako, Denmark) and against a mixture of the recombinant *B. burgdorferi* antigens p18, p41 internal fragment, p100 and outer surface protein C (OspC) (recomWell ELISA, Mikrogen, Germany). Both IgM and IgG antibodies to a synthetic *B. burgdorferi* C6-peptide were measured using a QuickELISA C6 Kit (Immunetics, MA, USA), followed by a Western blot (WB) (RecomBlot, Mikrogen, Germany) for analysis of IgG antibodies to recombinant *B. burgdorferi* antigens.

Allergic propensity was examined at baseline, by measuring total and allergen-specific IgE antibodies in plasma (ImmunoCAP™, Phadia AB, Sweden), according to the manufacturer's instructions. Specific IgE antibodies directed to common food and inhaled allergens were measured with Phadiatop Combi® (Phadia AB). A sample was considered positive, if at least one of the different assays was positive.

Before antibiotic treatment, a 4 mm skin punch biopsy was taken under local anesthesia (Xylocain 10 mg/ml, AstraZeneca, Sweden), from the outer edge of the red EM zone and from an opposite, healthy body site (control). The biopsies were split in two pieces and immediately snap-frozen in a mixture of isopentane and liquid CO_2_ and stored at −70°C. Serial sections (6 µm) were later obtained using a Leitz CM3050 cryostat (Leica Microsystems, Gothenburg, Sweden) and 2–6 sections were placed in each well of a three-well microscope slide (Novakemi, Stockholm, Sweden) pre-coated with 0.1% poly-L-lysine (Sigma, Stockholm, Sweden). These preparations were air-dried and kept at −70°C, until stained.

Due to the complicated and time-consuming laboratory procedure, not all biopsies from the asymptomatic patients were used for immunohistochemical staining. Thus, cytokine expression was analysed in biopsies from the 7 patients with and 18 (originally chosen to match for age and sex) without symptoms at the 6-month follow-up. These patients were considered representative of the total asymptomatic group (n = 81) in terms of reported symptoms at baseline and at the three month follow-up (data not shown). The slides were fixed for 5 minutes at 4°C in an acetone bath and then dried for 30 min at room temperature (RT), after which endogenous biotin was blocked using a streptavidin/biotin blocking kit (Immunkemi AB, Sweden). Saponin-phosphate buffered saline (PBS, 0.1%; Sigma-Aldrich AB and Medicago AB, Sweden) was used as washing and dilution buffer. Before incubation, the slides were washed for 30 min in 5% normal serum (containing goat, mouse and rat serum, each at 5%; [code numbers X0907, X0910 and X0912; DakoCytomation, Denmark]). Antibodies were titrated for maximal fluorescence in sections of inflamed tonsil [42] and validation of the staining method was done using isotype antibodies and PBS as negative controls. The slides were incubated in a humidified chamber for 60 min at RT with the following antibodies: primary mouse anti-human interferon (IFN)-γ monoclonal IgG1 (diluted 1∶1000, clone 1-D1K; Mabtech AB, Sweden), mouse anti-human interleukin (IL)-12p70 monoclonal IgG1 (1∶500, clone 24945; R&D Systems, MN, USA), rat anti-human IL-4 monoclonal IgG1 (1∶500, clone MP4-25D2; BD Biosciences, CA, USA), rat anti-human IL-10 monoclonal IgG2a (1∶500, clone JES3-19F1; BD Biosciences) and isotype mouse-IgG1κ antibodies (1∶1000 for IFN- γ and 1∶500 for IL-12p70, clone MOPC-21; BD Biosciences), rat-IgG1 (1∶500, clone R3-34; BD Biosciences) and rat-IgG2a (1∶500, clone R35-95; BD Biosciences). After washing, the slides were further incubated for 60 min at RT with secondary antibodies; goat anti-mouse polyclonal IgG1 conjugated with Cy3 (diluted 1∶10 for IFN- γ and 1∶100 for IL-12p70; Caltag Laboratories, Burlingname, CA, USA) and goat anti-rat IgG F(ab')_2_ conjugated with fluorescein (FITC, diluted 1∶10 for IL-4 and 1∶100 for IL-10; Jackson ImmunoResearch Laboratories Inc., West Grove, PA, USA). After additional washing, a drop of SlowFade®Antifade mounting solution (Molecular Probes, Eugene, OR, USA) was added to each well, cover glasses were mounted and the slides were stored at +4°C (maximum 48 h).

The slides were anonymised and all section were blindly analysed according to a protocol by two of the authors (JS and LF), using a Nikon Eclipse E600 (and Nikon EZ-C1 software version 3.30, Nikon Instruments Europe, the Netherlands) at excitation wavelengths of 546 (Cy3) and 488 (FITC) nm. Cytokine expression was estimated on a scale from 0 to +++; where +, ++ and +++ corresponded to 1–10, 10–20 and >20 cytokine expressing (positive) cells/section, respectively (Table 4). These estimations were given numeral values (0 = 0, + = 1, ++ = 2 and +++ = 3). Thus, a semi-quantitative and comparable estimation of the cytokine expression in the skin specimens was obtained.

**Table 4:** Estimation of cytokine expression in skin biopsies.

Scale	No. of cytokine expressing cells
0	0
**+**	1–10
**++**	11–20
**+++**	>20

The presence of IL-4, IL-10, IL-12p70 and IFN-γ in serum samples obtained at inclusion and plasma samples from the 3- and 6-month follow-ups was determined using a Milliplex™ MAP High Sensitivity Human Cytokine kit (HSCYTO-60SK; Millipore Corporation, Billerica, MA, USA) according to the manufacturer's instructions. STarStation v.3.0 (Applied Cytometry, Sheffield, UK) was used for data acquisition and analysis. Values below the lowest value of the standard curve were assigned half the values of the lowest standard point.

One half of each skin biopsy was analysed for the presence of *B. burgdorferi* DNA. DNA extraction and quantitative real-time polymerase chain reaction (PCR) was performed as described by Comstedt *et al.* [43]. DNA was extracted using the Puregene DNA isolation protocol (Gentra Systems, Minneapolis, MN, USA) and stored at −20°C. DNA extracts were analysed for presence of the *B. burgdorferi* complex by a quantitative PCR assay with probes and primers specific for the 16S rRNA gene. Serially diluted *B. burgdorferi* 31 and *B. hermsii* HS1 DNA were used as standards. *B. burgdorferi* species were identified by direct sequencing of the amplicons generated from the /rrs/(16S)-/rrl/ (23S) intergenic spacer or 16S gene PCRs. When necessary, nested modification of these assays was used to increase amplification success. Skin biopsies (92 EM and 87 control biopsies) were analysed using quantitative real-time PCR for detection of *B. burgdorferi* DNA. Biopsies found to contain *B. burgdorferi* DNA were analysed by 16S PCR alone or 16S PCR followed by an OspC-nested PCR. The biopsies that were positive in the OspC-nested PCR were sequenced with OspC-primers. 16S primers were used to increase accuracy if results obtained with the OspC-primers were uncertain. All negative OspC-nested analyses were sequenced with 16S-primers.

### Statistical analyses

Clinical data and *B. burgdorferi*- and allergen antibody responses were analysed using logistic regression, Fisher's exact test or Mann-Whitney U-test for comparison of the two groups (*ie.* patients with and without symptoms at the 6-month follow-up). Mann-Whitney U test was used to compare the two groups regarding cytokine expression in skin biopsies and cytokine levels in serum and plasma. After Mann-Whitney, manual Bonferroni correction was performed to correct for the four cytokine variables. Wilcoxon signed rank test was used to compare the cytokine expression in EM lesions with the corresponding expression in unaffected skin. Fisher's exact test was applied to serum and plasma data, since they included many values below the detection level and additionally for analysis of the distribution of detectable DNA of different *B. burgdorferi* subspecies. Spearman's rank correlation test was used to assess associations between cytokine expression in the skin biopsies and blood samples. Statistical analyses were performed using PASW Statistics 18 software for Microsoft (SPSS Inc., Chicago, IL, USA) and GraphPad Prism version 5.02 for Microsoft (GraphPad Software Inc, San Diego, CA, USA). A p-value≤0.05 was considered significant, whereas a p-value≤0.0125 was considered significant after correction for multiple variables.
